# In Regard to Kılıç et al.

**DOI:** 10.4274/balkanmedj.galenos.2019.2019.5.43

**Published:** 2019-07-11

**Authors:** Muhammed Keskin, Ahmet Lütfullah Orhan

**Affiliations:** 1 Clinic of Cardiology, Health Sciences University, Sultan Abdulhamid Han Training and Research Hospital, İstanbul, Turkey

To the Editor,

We read with interest the paper by Kılıç et al. (1) entitled “The Prevalence and Risks of Inappropriate Combination of Aspirin and Warfarin in Clinical Practice: Results From WARFARIN-TR Study”. The authors evaluated the inappropriate use of aspirin in patients with warfarin treatment, and a combination of aspirin and warfarin treatment was defined as an inappropriate treatment in patients with mechanical heart valve. We have some comments about this study.

The latest American College of Cardiology/American Heart Association guideline for the management of patients with valvular heart disease recommends aspirin 75 to 100 mg daily in addition to anticoagulation with warfarin in patients with mechanical valve prosthesis (2). Turpie et al. (3) studied patients with heart valve replacement and reported that the addition of aspirin to warfarin therapy reduced mortality and major systemic embolism in patients with mechanical heart valves and high-risk patients with prosthetic tissue valves. Although there was some increase in bleeding, the risk of combined treatment was more than offset by the considerable benefit (3).

The latest American College of Chest Physicians guideline for antithrombotic and thrombolytic therapy recommends the addition of low-dose aspirin (50-100 mg/day) to warfarin therapy in patients with mechanical heart valves at low bleeding risk (4). The 2017 European Society of Cardiology and the European Association for Cardio-Thoracic Surgery guideline for the management of valvular heart disease does not indicate the combination therapy of warfarin with aspirin as a contraindication, although it reported that the addition of lower dose antiplatelet agents (e.g., aspirin 75-100 mg/day) might be reserved for specific indications. Additionally, this guideline reported that the addition of low-dose aspirin to warfarin should be considered after thromboembolism despite adequate and inadequate international normalized ratio (5). However, in the WARFARIN-TR study, the authors reported that it is not clear how many patients had acute ischemic attack or thromboembolism during the preceding year.

Consequently, the addition of low-dose aspirin to warfarin therapy is widely considered as an antithrombotic therapy in patients with mechanical heart valves, and none of the guidelines contraindicated this combination therapy. Therefore, the definition of “inappropriate combination therapy” might not be appropriate in patients with mechanical heart valves.
